# A spherical-plot solution to linking acceleration metrics with animal performance, state, behaviour and lifestyle

**DOI:** 10.1186/s40462-016-0088-3

**Published:** 2016-09-23

**Authors:** Rory P. Wilson, Mark D. Holton, James S. Walker, Emily L. C. Shepard, D. Mike Scantlebury, Vianney L. Wilson, Gwendoline I. Wilson, Brenda Tysse, Mike Gravenor, Javier Ciancio, Melitta A. McNarry, Kelly A. Mackintosh, Lama Qasem, Frank Rosell, Patricia M. Graf, Flavio Quintana, Agustina Gomez-Laich, Juan-Emilio Sala, Christina C. Mulvenna, Nicola J. Marks, Mark W. Jones

**Affiliations:** 1 Swansea Lab for Animal Movement, Biosciences, College of Science, Swansea University, Singleton Park, Swansea, SA2 8PP UK; 2 Visual Computing, Computer Science, College of Science, Swansea University, Singleton Park, Swansea, SA2 8PP UK; 3 School of Biological Sciences, Institute for Global Food Security, Queen’s University Belfast, Medical Biology Centre, 97, Lisburn Road, Belfast, BT9 7BL UK; 4 Institute of Life Science, Swansea University Medical School, Swansea, SA2 8PP UK; 5 Centro Nacional Patagonico, Boulevard Brown s/n, Chubut, Argentina; 6 Applied Sports Science Technology and Medicine Research Centre, College of Engineering, Swansea University, Fabian Way, Swansea, SA1 8EN UK; 7 Department of Biological and Environmental Sciences, College of Arts and Sciences, Qatar University, Doha, 2713 Qatar; 8 Faculty of Arts and Sciences, Department of Environmental and Health Studies, Telemark University College, N-3800 Bø i, Telemark, Norway; 9 Department of Integrative Biology and Biodiversity Research, Institute of Wildlife Biology and Game Management, University of Natural Resources and Life Sciences, Vienna, A-1180 Vienna Austria

**Keywords:** Spherical plots, Tri-axial acceleration, G-sphere, Visualisation

## Abstract

**Background:**

We are increasingly using recording devices with multiple sensors operating at high frequencies to produce large volumes of data which are problematic to interpret. A particularly challenging example comes from studies on animals and humans where researchers use animal-attached accelerometers on moving subjects to attempt to quantify behaviour, energy expenditure and condition.

**Results:**

The approach taken effectively concatinated three complex lines of acceleration into one visualization that highlighted patterns that were otherwise not obvious. The summation of data points within sphere facets and presentation into histograms on the sphere surface effectively dealt with data occlusion. Further frequency binning of data within facets and representation of these bins as discs on spines radiating from the sphere allowed patterns in dynamic body accelerations (DBA) associated with different postures to become obvious.

**Method:**

We examine the extent to which novel, gravity-based spherical plots can produce revealing visualizations to incorporate the complexity of such multidimensional acceleration data using a suite of different acceleration-derived metrics with a view to highlighting patterns that are not obvious using current approaches. The basis for the visualisation involved three-dimensional plots of the smoothed acceleration values, which then occupied points on the surface of a sphere. This sphere was divided into facets and point density within each facet expressed as a histogram. Within each facet-dependent histogram, data were also grouped into frequency bins of any desirable parameters, most particularly dynamic body acceleration (DBA), which were then presented as discs on a central spine radiating from the facet. Greater radial distances from the sphere surface indicated greater DBA values while greater disc diameter indicated larger numbers of data points with that particular value.

**Conclusions:**

We indicate how this approach links behaviour and proxies for energetics and can inform our identification and understanding of movement-related processes, highlighting subtle differences in movement and its associated energetics. This approach has ramifications that should expand to areas as disparate as disease identification, lifestyle, sports practice and wild animal ecology.

UCT Science Faculty Animal Ethics 2014/V10/PR (valid until 2017).

**Electronic supplementary material:**

The online version of this article (doi:10.1186/s40462-016-0088-3) contains supplementary material, which is available to authorized users.

## Background

Quantification of animal movement is a hugely complex topic. In its broadest sense, it operates over wide (3-dimensional) space-scales and highly variable time periods. For example, it encompasses everything from a single limb motion describing a simple arc lasting less than a second, through co-ordination of repetitive limb motion in a whole animal during travel, which may last hours, to the diversity in the complex movement describing the various behaviours exhibited over the lifetime of an animal. Understanding animal movement is important for a suite of reasons but particularly because voluntary animal movement requires energy. Quantification of the allocation of chemical energy for mechanical output and how this relates to movement is relevant in understanding the costs, efficiencies and values of behaviour, lifestyle and exercise physiology. Judicious use of energy is a major element of optimization studies that seek to define best strategies, which have a broad remit ranging from examining most enhanced performance by elite athletes [1, 2] to animals adopting behaviours that maximize survival [3].

Unsurprisingly, therefore, the energetics of movement is well studied e.g. [4], but it has been polarised into essentially two main branches defined by differing methodologies - One branch examines power use [5], which typically requires measurement across extended periods [6–8] but is limited by the difficulties in attributing instantaneous power to performance [9]. The other seeks to quantify behaviour, relying variously on approaches such as high-speed cameras [10], point light displays [11] and force platforms [12] for work on humans and, primarily, on observation-based methodologies for wild animals [13].

Increasingly though, both the power use and the behaviour of humans [14] and animals [15] are being studied using accelerometers in animal/human-attached tags because these sensors quantify change in speed, a fundamental property of motion, precisely [16]. Thus, in the field of energetics, workers have derived indices, such as those based on dynamic body acceleration (DBA) metrics [17], that correlate tightly with oxygen consumption [18], while behavioural studies have used various methods such as random forests, vector machines and artificial neural networks on acceleration data to identify behaviours [19, 20]. However, both groups recognise the problem inherent in the complexity of acceleration data. These provide most value when recorded at high rates (typically >20 Hz) across each of the three axes defining orientation in space, producing effectively 6 channels of data, 3 relating to the gravity-based component of the acceleration and 3 relating to the animal-based movement [21]. Indeed, it is perhaps this complexity that still represents an appreciable challenge for the animal (and human) behaviour community in binding energy use and behaviour within one framework (cf. [22]), even though they are fundamentally interdependent. Indeed, any framework that enhances consideration of animal movement, behaviour and power use simultaneously should facilitate the identification and understanding of processes and patterns across and between them.

One solution to this is to recognise that, because the earth’s gravity is constant, a tri-axial plot of tri-axial, orthogonally placed, acceleration data fundamentally builds a sphere, a ‘g-sphere’ [23]. Acceleration data derived from animal movement change the form of this sphere. We capitalise on this to create a new visualization paradigm for animal/human-attached acceleration data whereby we generate the g-sphere and then place animal movement data on it, including those that seek to exemplify power use, over any temporal scale. The approach marries behaviour to estimated energetics and highlights some patterns that are not intuitively obvious. We show that g-sphere visualizations should have the capacity to highlight changes in movement patterns associated with e.g. human emotional state, injury and best practice in single sports manoeuvres but extend through to highlighting proxies for energy-based behavioural ecology in wild animals over time periods ranging from seconds to years.

## Results

### The basic g-sphere

**Fig. 1:**
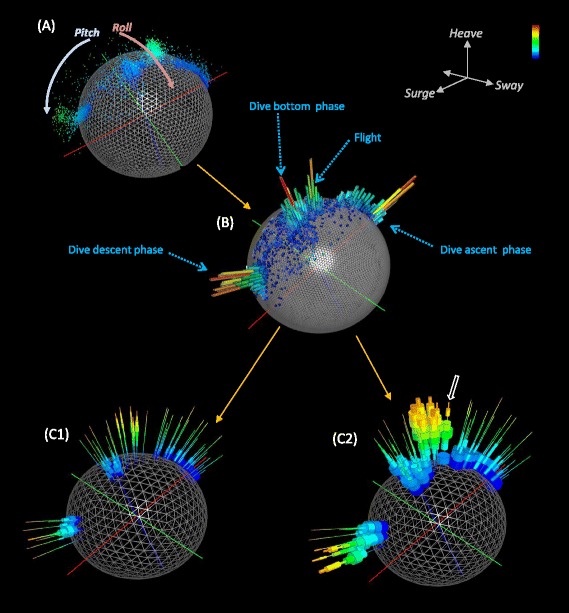
Example behavioural data from a cormorant. Six dives and a short period of flight are visualised by (**a**) a point- based g-sphere [with point colour equating with DBA]. **b** shows the same data as (**a**) but as a Dubai plot. Both (**c**) images depict urchin plots of (**b**); *C1* shows percentages of DBA allocation taken across the whole g-sphere while *C2* shows percentages amounting to 100 % per facet. Note the higher values of DBA attributed to flight and descent of the water column, particularly emphasized by the 100 % facet percentage. Note also how certain spines show multi-modes (e.g. *white arrow*) which can be indicative of different behaviours at one body attitude

An animal-attached tag mounted in the centre of an animal’s back with orthogonal, tri-axial accelerometers (aligned with the major axes of the body) produces a ‘static’ g signal with a vectorial sum of 1.0 *g* due to gravity when the animal is stationary. Plots of such tri-axial data in a 3-d graph therefore tend to populate the outer surface of the g-sphere which becomes most apparent as the animal adopts body orientations with multiple combinations of body pitch and roll (Fig. 1a). When animals move, points may leave the g-sphere surface as acceleration values reflect g-forces derived from the animal’s acceleration (Fig. 1a). This has been termed ‘dynamic acceleration’ and can be dealt with in a number of different ways, one of which is to remove it by selective smoothing [21] and normalising (see methods) to leave the postural data. Thus, body attitude, which is a major step in elucidating behaviour [16], is defined by the position of the data points on the sphere.

### Dealing with over-plotting - the Dubai plot

**Fig. 2:**
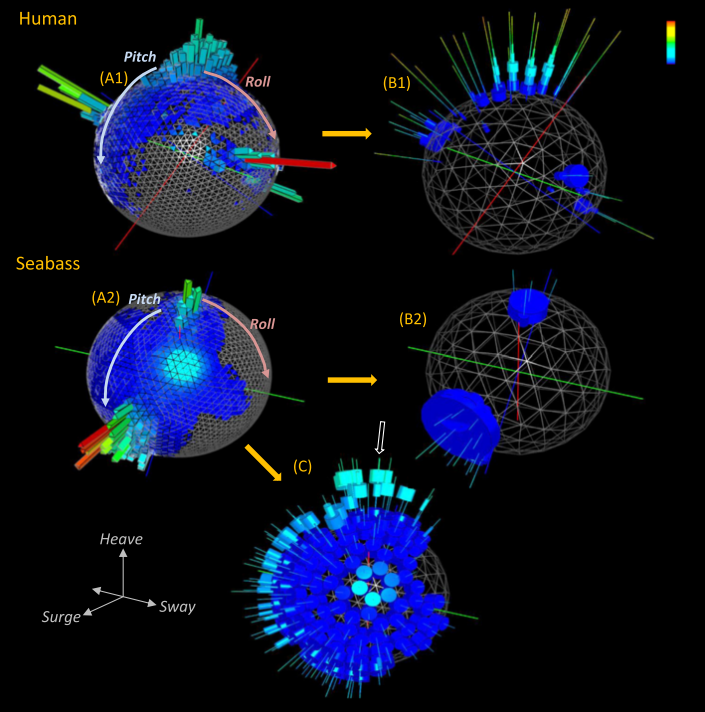
Examples of posture and energy-linked posture visualised for two contrasting species (a human and a fish) over 24 h. The human data are taken from a person on a walking/camping tour while the fish data are from a hole-dwelling reef species that often rests by wedging itself at unusual angles. The left hand figures (**a**) show spherical histogram (Dubai) plots, indicating how time is allocated to different body postures [the ‘North pole’ position shows the species in the ‘normal’ upright position]. The first right-hand figure for each species (**b**) shows how each posture is linked to varying putative power levels. Note how the human has higher power-proxy levels associated with the vertical posture due to walking. Both the human and the fish have low power-proxy levels at low ‘latitude’ angles acquired during resting/sleep, exemplified by the large diameter blue discs. Data normalized to give a global percentage for all angles may hide infrequent, but higher-energy, activities. Normalising the data to 100 % per facet (**c**) highlights these though. In this case, the low-energy life style of the fish is still apparent (cf. B), with higher energies occurring fleetingly and only when the fish is vertical (*white arrow*). The colour coding has blue as low, and red as high, values

Increasing time periods viewed within the basic g-sphere tend to result in increasing occlusion and over-plotting of the data, making visualizations more confusing and less useful as the number of data points increases (Fig. 1a). A representation of the time allocated to various postures can, however, be obtained by tessellating the surface of the *g*-sphere into facets, summing the data points within each facet, and presenting the number of points within each facet by a projection into space away from the g-sphere, producing a spherical histogram or ‘Dubai plot’ (Fig. 1b). Such plots typically show modes representing different type of behaviour with the higher peaks representing the more common behaviours (Figs. 1b and 2a).

### Allocating putative power use to the g-sphere - the g-urchin

**Fig. 3:**
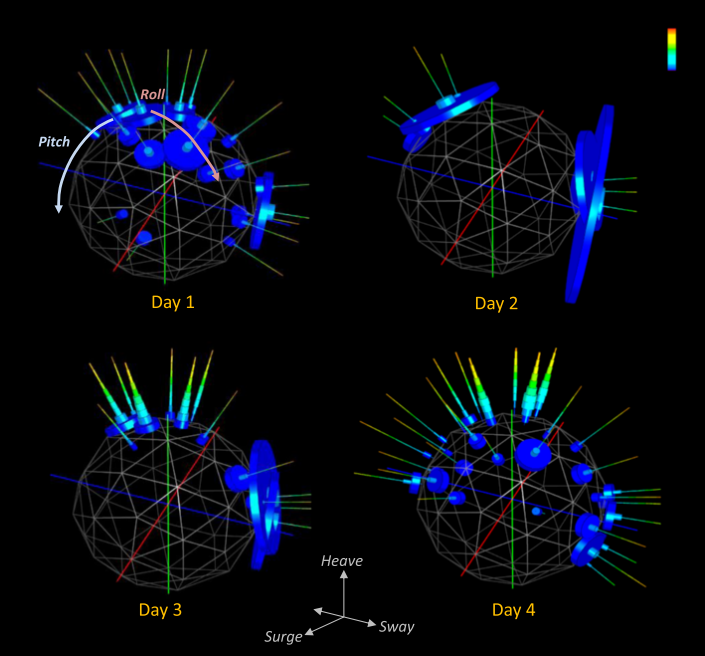
Example urchin plots for four consecutive 24 h periods after the release of a European badger (wearing a collar-mounted accelerometer) following anaesthesia. The ‘North pole’ facets show when the animal was properly horizontal (ie in standing or walking posture). Note how the first two days show no high energy activity because the animal was either resting or asleep. The second day shows only four changes in position. By day three, higher energy, normal posture activities such as walking are apparent at the North pole. This process is further enhanced in day 4, with North pole spine DBA distributions having modes that have moved up the length of the spines to indicate higher power use. DBA values are colour-coded with maximum values (in *red*) of 1 *g*

While basic g-spheres and Dubai plots quantify the time allocated to different postural states, they impart no information on power use. This information can be incorporated into the g-sphere by calculating the dynamic body acceleration (DBA) (see methods), which correlates linearly with power [18], for each of the postural data points within each facet on the sphere. In this, we note that although one study has shown that a strong relationship between DBA and energy expenditure holds for a (seabird) species operating in three media and multiple different body angles [22], confirmation that this is also the case for more species will need further work (but see [24]). To visualize this, the sphere facets can be populated with thin spines, one spine per facet, radiating into space, like a sea urchin (facets without data have no spine). Spines acquire stacked rings representing the frequency distribution of the DBA values associated with that posture/facet. The position of each ring on the spine indicates the DBA value (lower values are closer to the g-sphere surface), the depth of the ring indicates the width of the DBA bin, and the diameter of the ring is proportional to the number of data points within that bin (Figs. 1c and 2b). This ‘g-urchin’ can be represented so that it is normalised for all data across the sphere, which highlights the processes that dominate in terms of both the time and proxy for energy across the whole time period considered (Figs. 1c1 and 2b). Alternatively, data can be normalised within each facet to highlight the energetic proxies of particular postures irrespective of their time contribution (Figs. 1c2 and 2c). Urchin plots thus show differences between behaviours within species (Fig. 1c), differences in lifestyles between species (Fig. 2b, c), and differences in behaviour of any individual through time (Fig. 3).

### Comparing behaviours and putative power uses - the differential g-urchin

**Fig. 4:**
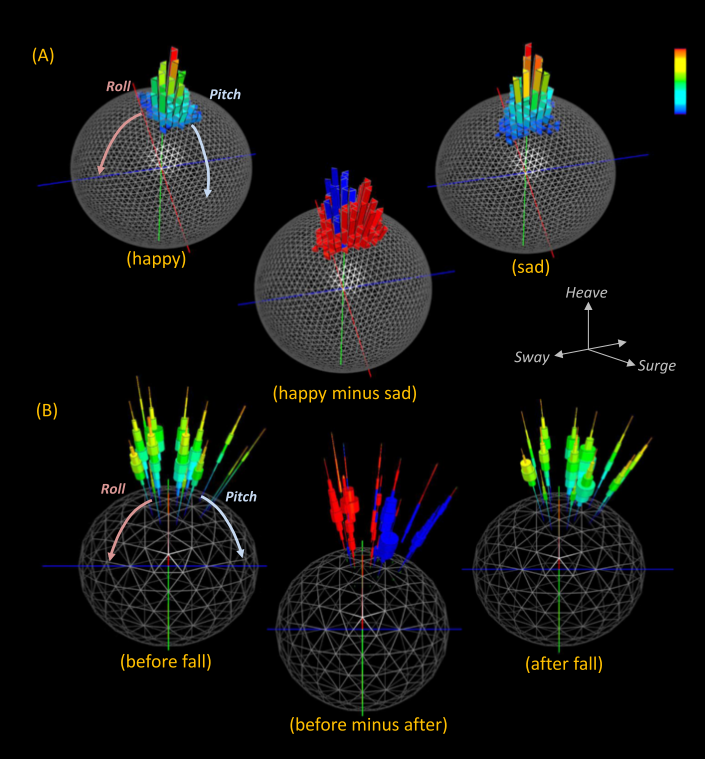
Example posture and DBA values associated with ‘state’ in humans. **a** shows two Dubai plots for a person walking after seeing ‘happy’ and ‘sad’ film clips (higher frequencies are coded by warmer colours). A third differential Dubai plot highlights the difference between the two situations (*blue* = a higher relative frequency of ‘happy’ points per facet while red = a higher relative frequency of ‘sad’ points per facet). Note how the two conditions are reflected in the postural changes (**b**) shows urchin plots for someone trekking across snow pulling a sledge one minute before a fall and one minute after recovering from the fall. The differential urchin shows both differences in postures adopted between the two situations as well as the dynamism of the walking (red shows a higher relative DBA frequency ‘before the fall’ while blue shows the reverse)

The process of comparing individuals or the same individual over different times can be enhanced by subtracting one Dubai plot or one g-urchin from another. These differential plots can be colour-coded, for example, according to which DBA bin from which urchin has the higher value (Fig. 4). This highlights differences in assumed power use associated with posture and therefore behaviour, with notable changes even associated even with state [25] (Fig. 4a).

### Simplifying outputs

**Fig. 5:**
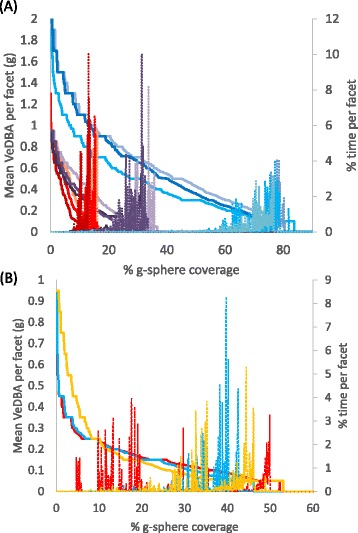
Example ‘lifestyle’ plots for different species and situations. These show how DBA values are distributed across the surface of the g-sphere (*continuous lines*) and the time allocated to those values (*dashed lines of equivalent colour*) over 24 h for (**a**) 3 Magellanic penguins (*blue*), 3 Eurasian beavers (*purple*) and 3 domestic sheep (*red*) and (**b**) three people; a child (*yellow*) and 2 adults, one of whom hiked extensively during the period (*red*) while the other was essentially sedentary (*blue*). Note the species-specific similarities (species that employ most diverse body angles have the highest percentage of the sphere coverage) but that differences between individuals can be manifest in either the time or DBA allocations on the sphere

G-sphere derivatives can be re-simplified to enhance e.g. inter- or intra- specific comparisons by plotting 2-d line graphs showing the time and/or the DBA allocated to percentage coverage of the g-sphere (Fig. 5). Such ‘lifestyle’ plots show consistent patterns within and between species (Fig. 5).

## Discussion

Application of g-spheres and their derivatives to raw tri-axial acceleration data adds another powerful tool to visualize and identify behaviour [19] that requires no knowledge of the animal in question for behaviour-specific patterns to emerge into groups. This approach concatenates 6 complex lines of acceleration data into one plot binding animal attitude and proxy for power use into one visualization that clearly shows modes of behaviour (Fig. 1). The immediate value lies in its potential for use as a template match approach for specific activity pattern identification across data [26]. Thus, behavioural description and identification (Figs. 1, 2 and 3) do not require matched observed behaviours with example data but stem from a visually apparent clustering within the plot. In particular, differences between various g-sphere derivatives, especially Dubai and urchin plots (Fig. 4), can be used to identify specific variation in posture and power-use proxies between behaviours. For example, the Dubai plots in Fig. 4a provide an example of how the posture of a subject changed according to whether they had watched a happy or sad film clip, with the allocation of time to facet position changing. Similarly, the posture and allocation of DBA to different body postures during walking changed after a fall (Fig. 4b). The g-spheres therefore employ fundamentally different principles to other methods in the manner of data visualization and interpretation.

In a first iteration, the most common behaviours are most easily identified because of the way they dominate the basic g-sphere visualization (Fig. 1), which could be argued is the most important feature of understanding time management in animals. However, even behaviour that is only a small fraction of the time budget, but is energetically distinct and therefore likely to be apparent in the DBA distributions on urchin spines, may be identified by moving from the globally normalized g-urchin to one that is normalized to facet (Fig. 2b, c).

Importantly, mono-, bi-, or even tri-modality in the frequency distributions of DBA allocated to particular facets or groups of adjacent facets, point to multiple behaviours occurring at similar animal postural attitudes. This is illustrated, for example, in the cormorant behaviour where the white arrow in Fig. 1c2 shows multi-modality in DBA due to both dive ascent behaviour and flight behaviour being apparent in the same body attitude facet. It is also exemplified in the stationary and swimming behaviours in the seabass, shown in the bimodality of the DBA distributions along urchin spines at the North Pole (cf. Fig. 2c). The time-based adoption of behaviours can also be studied with this, for example, in the badger data presented (Fig. 3). Here, ‘normal’ walking behaviour is only manifest during day 4 post-sedation, when the urchin spines at the North Pole acquire a DBA mode that is greater than 1.0 *g* (Fig. 3). Such observations can then readily be incorporated into statistical classifiers and classification algorithms.

Generation of frequency distributions of DBA, as a proxy for power, thus enhances the process of separating behaviours. Importantly, it also helps visualize the overall allocation of power proxies, either to specific behaviours over short periods such as seconds or to collections of behaviour over longer periods (cf. Figs. 1, 2 and 3) extending to months or even years. Depending on the timescales, collections of particular behaviours should provide a representation of different lifestyles, as well as their considered associated energetic outlay, allowing powerful comparisons to be made between systems or scenarios. Examples include comparisons between species with contrasting lifestyles (Figs. 2 and 5) or within-species lifestyle comparisons. Indeed, the precise form of ‘lifestyle’ plots (Fig. 5) may help in defining lifestyle taxa by defining animal capacities. The future may also benefit from the use of g-sphere approaches based on multiple accelerometers used on different parts of the body or even having accelerometers on hand-held objects. The expectation is that this will be particularly useful in sport applications (Additional file 1: Figure S7) where effective movement must be stylized for maximum performance because limb-, or sports equipment-mounted sensors will represent local forces and perhaps local power-usage proxies better than trunk-mounted systems which produce a body-integrated signal. Importantly, such power-proxy comparisons, from trunk-or limb-mounted sensors, can help identify efficient solutions to activities where performance, such as running speed over a given distance or animal breeding success over months, should be equatable with the putative energetic cost. This sort of consideration thus has advantages for elite athletes as well as for conservation bodies examining the costs of the lifestyle of their animals. Equally, changes in behaviour that occur with disease or illness, such as constrained activity stemming from rheumatoid arthritis [27], should be rapidly identifiable using this approach.

We expect g-spheres and their derivatives (e.g. Fig. 5) to form the basis for summary statistics which highlight particular aspects of performance, behaviour and lifestyle, which may function to be powerful descriptors of e.g. animal lifestyle, linked, among other things, to physical limitations based on taxonomic, allometric or environmental (e.g. water versus terrestrial) constraints [28]. In addition, such visualizations may help both children and adults to understand how the physical activity levels in their lifestyles compare to those recommended [29].

## Conclusions

The treatment of tag-derived tri-axial acceleration data by creating a tri-axial plot of the gravity-based acceleration, leads to a spherical surface on which acceleration proxies for power use can be placed. This process has potential for highlighting behaviour-, and even state-, dependent clusters, irrespective of whether the user has a verified library or not and should illustrate how animals may allocate energy to the different behaviours. Subsequent simplification of the spherical plots into percentage of sphere occupied, mean dynamic body acceleration and time allocated per facet allows simple 2-d plots between these parameters to be created (Fig. 5). This approach should provide a powerful summary of putative energy allocation to behaviour and time, documenting intra-specific differences and showing how animals respond to their environment over time. Inter-specific comparisons of these metrics show promise as a powerful behavioural tool with which to compare and quantify animal lifestyles.

## Methods

The g-sphere visualization technique has been incorporated into publically available smart sensor analysis software, Framework4 [30, 31], available from http://www.framework4.co.uk. Walker et al. [32] give more details on this.

### The basic g-sphere

In brief, the basic g-sphere is derived from tri-axial acceleration data, where the sensors have orthogonal placement, aligning with the major axes of the tagged animal’s body.

Typically, the acceleration data will be recorded at infra-second rates (e.g. 40 Hz) on a deployment spanning anywhere from a few minutes up to a year. One day of data (24 h) recorded at these rates provides over 10 million measurements. For the g-sphere, we build on a method for visualising accelerometer data in Grundy et al. [23], using spherical coordinate plots to depict the distributions of data. To deal with large datasets, we utilise frequency-based approaches which show an overview of the data. Firstly, a spherical histogram shows the number of data items in each facet of the spherical coordinate system. Secondly, we build on the surface provided by the g-sphere using location-dependent frequency bins (the ‘g-urchin’ plot), for metrics such as DBA [17] as proxies for power usage. Multiple urchins can be compared difference operations to analyse across instances, behaviours groupings, or data sets.

### Static and dynamic acceleration

Measured acceleration is the product of a static component due to gravity, manifest in accelerometers according to their orientation with respect to the Earth, and a dynamic component, due to the movement of the animal. Separating these components from the raw accelerometer measurements allows isolation of postural attitudes and movement.

The static component can be approximated by applying a low-pass filter over each the accelerometer axis components. Shepard et al. [21] suggest smoothing using a running mean over a period amounting to about twice the wavelength of any repetitive frequencies. The static component at data point *i* (*SA*_*c*_*,i*) with a smoothing window of *w* is given by:

$$ {S}_i = \frac{1}{w}\ {\displaystyle \sum_{j=i-\frac{w}{2}}^{i + \frac{w}{2}}}{A}_j $$

The corresponding dynamic components of acceleration (*DA*_*c*_) per orthogonal axis are computed by subtracting the static components (*SA*_*c*_*)* of acceleration from the raw acceleration values (*A*_*c*_).

$$ D{A}_c = {A}_c - S{A}_c $$

### Power metrics

Dynamic acceleration-based metrics [17] have been argued as a predictor of power [18]. Two measures, Overall Dynamic Body Acceleration (ODBA) and Vectorial Dynamic Body Acceleration (VeDBA), have been used, and are essentially equivalent in terms of their power to predict VO_2_ [33].

VeDBA (*V*) is calculated from the dynamic components of acceleration (*DA*_*x*_, *DA*_*y*_ and *DA*_*z*_) by taking the vectorial length of the dynamic acceleration vector using;

$$ V = \sqrt{D{A}_x^2+D{A}_y^2+D{A}_z^2} $$

ODBA (*O*) is also calculated from the dynamic components of acceleration (*DA*_*x*_, *DA*_*y*_, and *DA*_*z*_), instead taking the sum of the dynamic acceleration components using;

$$ O = \left|\ D{A}_x\ \left|+\right|D{A}_y\ \right|+\left|D{A}_z\right| $$

### Raw plot

The basic g-sphere plots the static accelerometer data in a three dimensional scatter plot with the animal’s heave axis being allocated the y-axis, the surge the x-axis and the sway the z-axis (Fig. 1a). Each vector is considered as an offset from the origin, directly scatter-plotted in three-dimensional space with, for example, the colour of each data point being linked to any associated attribute in the data set (Fig. 1a). This representation shows short-lived behaviours well, providing a compelling visualization of when forces exceed that exerted by gravity (Additional file 1: Figure S1).

### Spherical plot

Normalising the static acceleration vector, encodes posture information. Given the *x*, *y*, and *z* channels of the vector, the length of the vector *L* can be computed and the components normalised to x’, y’ and z’ via:

$$ L = \sqrt{SA{X}^2+SA{Y}^2+SA{Z}^2} $$

$$ {X}^{\hbox{'}}=\frac{SAX}{L}\ {Y}^{\hbox{'}}=\frac{SAY}{L}\ {Z}^{\hbox{'}}=\frac{SAZ}{L} $$

This, projects the normalised vector onto the surface of a sphere in 3-d scatter plots which gives an implicit conversion to spherical coordinates (*r*, *θ*, *φ*) [34], where *θ* corresponds to the angle of inclination, *φ* is the angle of rotation on a two-dimensional plane, and the radius is constant (*r* = 1) throughout. Each vector is plotted as a point in the display and the size and radius of each point can be adjusted by a fixed amount, to link it to an attribute in the data set. Each point can be joined together in chronological order to show the temporal ordering of the vectors as a path in the three-dimensional space (Additional file 1: Figure S2) so that the spherical scatter plot shows an intuitive summary of the geometric distribution of posture and direction. Linking the radius, r of each coordinate to another attribute allows additional dimensions, such as depth, to be encoded which, in this case, provides a compelling illustration of diving patterns along with the associated state (Additional file 1: Figure S2).

### Binning in three-dimensions

Large data set plots incur problems with occlusion and overplotting where data values in a point cloud obscure other values. For this, an overview and focus approach [35] can be employed which gives a contextual overview of the data while leaving potential to interact with further details in the data. Thus, we divide the surface of the sphere into facets (*sphere tessellation*) and treat the data within each facet to derive summary statistics (*binning*).

### Sphere tessellation

To represent the underlying data on which the chart is based accurately [36], we employ a frequency-based approach using regular bin sizes to summarise the data although construction a sphere from a series of uniform geometric primitives is a problem from the cartography domain [37]. The traditional method of constructing a sphere via lines of latitude and longitude results in variable sized facets misrepresenting the underlying data [38]. We thus utilise a geodesic sphere, providing a close to uniform and regular sphere tessellation, using subdivision surfaces and spherical projection of an icosahedron platonic solid. The geodesic sphere starts with an icosahedron. Each facet is then repeatedly subdivided a pre-defined number of times with each of the acquired points projected onto a sphere. This results triangular facets, each of which is of a close to regular shape and area. Despite a slight variation in size and shape of each facet, this has a negligible effect in reconstructing the underlying data [38].

### Binning data

Binning identifies the facet with which a data item intersects on the geodesic sphere. Teanby [38] propose a winding method which operates by linearly searching for an intersecting facet on the sphere which has a sum of angles with the test vector equating to 2π. Walker et al. [30] propose a more efficient method using the hierarchical structure of the geodesic sphere which operates in a similar manner to that of a search tree, dividing and dealing with the otherwise logarithmic complexity. The angle (*θ*) between the direction of the centre of each facet from the origin, (*w*) and the current vector (*v*) is computed using the dot product (below). The point is determined to be associated with the sphere facet with the smallest angle between them. This is recursively computed on the hierarchical structure until the lowest-level is reached.

$$ \theta = \frac{v\ .\ w}{\left|v\right|\ \left|w\right|} $$

For each facet, the following statistics are computed; (i) the number of data items intersecting each facet, (ii) the mean value of each data channel for the items in each facet and (iii) a frequency distribution of a user-defined data attribute consisting of a user-defined number of bins. The data for attributes (i) and (ii) are normalised so that the whole sphere adds up to 100 %. The distributions for (iii) are normalised locally which allows the creation of a histogram of each facet of the power usage occurring for a particular movement and postural state independent of the frequency of the underlying data in the facet (since the frequency equates to a percentage).

### Dubai plot

The binned data for each facet can be displayed as a single histogram projecting perpendicularly from its respective facet (Figs. 1b, 2a and 4a; Additional file 1: Figure S3). Each histogram length and colour is nominally proportional to the normalised sample size for the sphere facet (Additional file 1: Figure S4). This gives an overview of the data distribution over the sphere, illustrating the frequency of postures or movements in the data set. The colour may be encoded as any other data attribute, in addition to the normalised frequency, along with the length.

### G-urchin

In a final step of this method, the smoothed, tri-axial acceleration axes can be encoded in addition to the frequency of items in each facet. A histogram for each facet of the sphere is computed for the items residing in the facet, which can be combined in a manner that represents the power usage for each state. This ‘g-urchin’ has spines projecting from the sphere with each spine placed at a user-defined distance away from the sphere to avoid occlusion with any of the other layers of the visualization (although a line is drawn to the centre of the facet it represents). The length and width of each spine can be ascribed to any data attribute. It is most effective when the spine characteristics are linked to histogram frequency or the number of items residing in the facet (Figs. 1c, 2b,c and 3). Each spine consists of a number of stacks, the width of which corresponds to the histogram bin width (Additional file 1: Figure S4 overview):

### Differential g-sphere

The binning procedure standardizes the data for time to allow a sphere from one situation (species, individual, time period) to be applied with another, providing the g-spheres are of the same sphere tessellation and bin size. We use two operations for this; firstly subtraction, which is used for highlighting differences, and summation, which combines g-spheres together. This gives the notion of two sphere types; a data g-sphere generated from raw data, and an operation g-sphere, generated by applying an operation. The standardization process means that operations can be applied to any combination of the two g-sphere representations.

- Difference is used to subtract two g-spheres (*GA*, *GB*) from each other. The absolute difference between the two spheres, for each facet in the sphere (*f*), and each corresponding bin (b) in the frequency distribution is computed. The result is a new operation g-sphere which highlights the difference between *GA* and *GB* (Fig. 4b).

$$ G\hbox{'} = {\displaystyle \sum_{i = 0}^f}{\displaystyle \sum_{j=0}^b} Abs\left(G{A}_{ij} - G{B}_{ij}\right) $$

(b) Summation is used to combine two g-spheres together. The items in each bin are added together. The result is a new operation g-sphere which combines the spheres *GA* and *GB* together.

$$ G\hbox{'} = {\displaystyle \sum_{i=0}^f}{\displaystyle \sum_{j=0}^b} Abs\left(G{A}_{ij} + G{B}_{ij}\right) $$

Each frequency distribution is normalised to eradicate any bias towards data sets containing different number of data points. The effect in the frequency distribution is a percentage where each bin contributes towards a subset of the distribution. As such, the entire frequency distribution totals 100 %. When combining the distributions together by addition or subtraction, the result is the difference in percentage between the two histograms. Percentages of distributions are used to protect against bias resulting from the size of the underlying data.

## Additional file

Additional file 1: Methods. Changing shapes for frequency distributions. **Figure S1.** A 3-d scatter plot (g-sphere) of static (orthogonal) tri-axial acceleration data. **Figure S2.** A spherical coordinate’s visualization of (a) postural state plotted onto the surface of a sphere in three-dimensional space, (b) points joined together in chronological order, (c) projecting the data outwards from the sphere according to other parameters. **Figure S3.** A spherical histogram (Dubai plot) visualization to depict frequent postural states. **Figure S4.** Histogram, Frequency shape (stacked), fixed shape (skittle) from urchin plots. **Figure S5.** G-urchin of skittle shape and stacked frequency urchins emitted from the centre of each facet of the sphere. **Figure S6.** Overview of user interface for a program in which spherical plots can be created. **Figure S7.** G-spheres and comparable g-urchins derived from a rod-mounted tri-axial accelerometer showing fly-fishing visualisations. (DOCX 5289 kb)

## Abbreviations

DBA: Dynamic body acceleration
VeDBA: Vectorial dynamic body acceleration

## References

[1] Jones AM (1998). A five year physiological case study of an Olympic runner. Br J Sports Med.

[2] Krustrup P, Hellsten Y, Bangsbo J (2004). Intense interval training enhances human skeletal muscle oxygen uptake in the initial phase of dynamic exercise at high but not at low intensities. J Physiol.

[3] Hamilton WD (1970). Selfish and spiteful behaviour in an evolutionary model. Nature.

[4] Bleich S, Ku R, Wang Y (2011). Relative contribution of energy intake and energy expenditure to childhood obesity: a review of the literature and directions for future research. Int J Obes (Lond).

[5] Scantlebury DM (2014). Flexible energetics of cheetah hunting strategies provide resistance against kleptoparasitism. Science.

[6] Reilly JJ (2004). Total energy expenditure and physical activity in young Scottish children: mixed longitudinal study. Lancet.

[7] Arch J, Hislop D, Wang S, Speakman J (2006). Some mathematical and technical issues in the measurement and interpretation of open-circuit indirect calorimetry in small animals. Int J Obes (Lond).

[8] Trost SG, Loprinzi PD, Moore R, Pfeiffer KA (2011). Comparison of accelerometer cut-points for predicting activity intensity in youth. Med Sci Sport Exer.

[9] Bassey E, Short A (1990). A new method for measuring power output in a single leg extension: feasibility, reliability and validity. Eur J Appl Physiol Occup Physiol.

[10] Meur Y (2013). Spring-mass behaviour during the run of an international triathlon competition. Int J Sports Med.

[11] Nackaerts E (2012). Recognizing biological motion and emotions from point-light displays in autism spectrum disorders. PLoS One.

[12] Girard O, Millet G, Slawinski J, Racinais S, Micallef J (2013). Changes in running mechanics and spring-mass behaviour during a 5-km time trial. Int J Sports Med.

[13] Watanabe YY, Takahashi A (2013). Linking animal-borne video to accelerometers reveals prey capture variability. Proc Natl Acad Sci.

[14] Yang C-C, Hsu Y-L (2010). A review of accelerometry-based wearable motion detectors for physical activity monitoring. Sensors.

[15] Brown DD, Kays R, Wikelski M, Wilson RP, Klimley AP (2014). Observing the unwatchable through acceleration logging of animal behavior. Anim Biotelem.

[16] Shepard EL, et al. Identification of animal movement patterns using tri-axial accelerometry. Endanger Species Res. 2008;10.

[17] Wilson RP (2006). Moving towards acceleration for estimates of activity-specific metabolic rate in free-living animals: the case of the cormorant. J Anim Ecol.

[18] Halsey LG, Shepard EL, Wilson RP (2011). Assessing the development and application of the accelerometry technique for estimating energy expenditure. Comp Biochem Physiol A Mol Integr Physiol.

[19] Nathan R (2012). Using tri-axial acceleration data to identify behavioral modes of free-ranging animals: general concepts and tools illustrated for griffon vultures. J Exp Biol.

[20] Preece SJ, Goulermas JY, Kenney LP, Howard D (2009). A comparison of feature extraction methods for the classification of dynamic activities from accelerometer data. Biomed Eng IEEE Trans on.

[21] Shepard EL (2008). Derivation of body motion via appropriate smoothing of acceleration data. Aquat Biol.

[22] Elliott KH, Le Vaillant M, Kato A, Speakman JR, Ropert-Coudert Y (2013). Accelerometry predicts daily energy expenditure in a bird with high activity levels. Biol Lett.

[23] Grundy E, Jones MW, Laramee RS, Wilson RP, Shepard EL (2009). Computer Graphics Forum.

[24] Laich AG, Wilson RP, Gleiss AC, Shepard ELC, Quintana F (2011). Use of overall dynamic body acceleration for estimating energy expenditure in cormorants: does locomotion in different media affect relationships?. J Exp Mar Biol Ecol.

[25] Wilson RP (2014). Wild state secrets: ultra-sensitive measurement of micro-movement can reveal internal processes in animals. Front Ecol Environ.

[26] Bartlett R (2006). Artificial intelligence in sports biomechanics: new dawn or false hope?. J Sports Sci Med.

[27] Semanik P (2010). Assessing physical activity in persons with rheumatoid arthritis using accelerometry. Med Sci Sports Exerc.

[28] Demetrius L (2006). The origin of allometric scaling laws in biology. J Theor Biol.

[29] Active SAS (2011). A report on physical activity for health from the four home countries’ chief medical officers.

[30] Walker JS (2015). TimeClassifier: a visual analytic system for the classification of multi-dimensional time series data. Vis Comput.

[31] Walker J, Borgo R, Jones MW (2016). TimeNotes: a study on effective chart visualization and interaction techniques for time-series data. Vis Comput Graph IEEE Trans on.

[32] Walker JS (2015). Prying into the intimate secrets of animal lives; software beyond hardware for comprehensive annotation in ‘Daily Diary’tags. Mov Ecol.

[33] Qasem L (2012). Tri-axial dynamic acceleration as a proxy for animal energy expenditure; should we be summing values or calculating the vector. PLoS One.

[34] Aigner W, Miksch S, Schumann H, Tominski C (2011). Visualization of time-oriented data.

[35] Shneidermann B. The eyes have it: A task by data type taxonomy for information visualizations. Proceedings of the IEEE Symposium 1996;336-43.

[36] Tufte ER, Graves-Morris PR (1983). The visual display of quantitative information.

[37] Van Wijk JJ (2008). Unfolding the earth: myriahedral projections. Cartograph J.

[38] Teanby N (2006). An icosahedron-based method for even binning of globally distributed remote sensing data. Comput Geosci.

