# Supplementary material for: A spherical-plot solution to linking acceleration metrics with animal performance, state, behaviour and lifestyle
Source: Mov Ecol. 2016 Sep 23;4:22. doi: 10.1186/s40462-016-0088-3 (PMC5035456; doi:10.1186/s40462-016-0088-3)
Supplement: Additional file 1: — Methods. Changing shapes for frequency distributions. Figure S1. A 3-d scatter plot (g-sphere) of static (orthogonal) tri-axial acceleration data. Figure S2. A spherical coordinate’s visualization of (a) postural state plotted onto the surface of a sphere in three-dimensional space, (b) points joined together in chronological order, (c) projecting the data outwards from the sphere according to other parameters. Figure S3. A spherical histogram (Dubai plot) visualization to depict frequent postural states. Figure S4. Histogram, Frequency shape (stacked), fixed shape (skittle) from urchin plots. Figure S5. G-urchin of skittle shape and stacked frequency urchins emitted from the centre of each facet of the sphere. Figure S6. Overview of user interface for a program in which spherical plots can be created. Figure S7. G-spheres and comparable g-urchins derived from a rod-mounted tri-axial accelerometer showing fly-fishing visualisations. (DOCX 5289 kb) [file 40462_2016_88_MOESM1_ESM.docx]

**Supplementary Information**


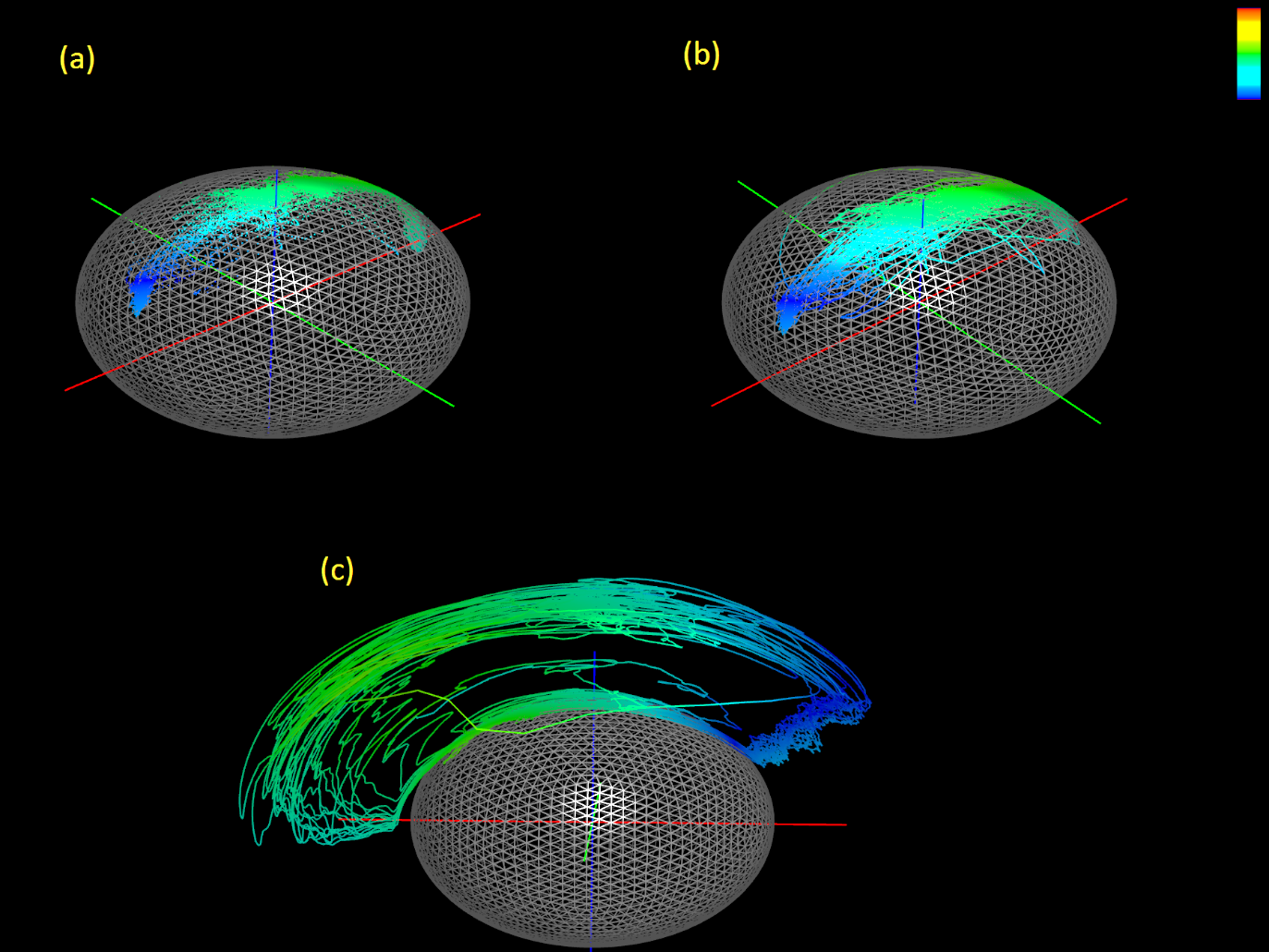


(A)

(B)

(C)


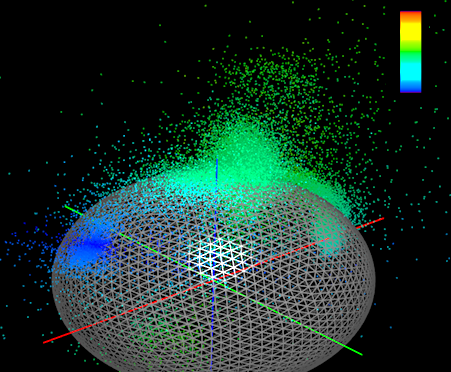


***Fig. S1*** *– A 3-d scatter plot (g-sphere) of static (orthogonal) tri-axial acceleration data recorded from an Imperial Cormorant; diving, sitting on the water surface and flying.*


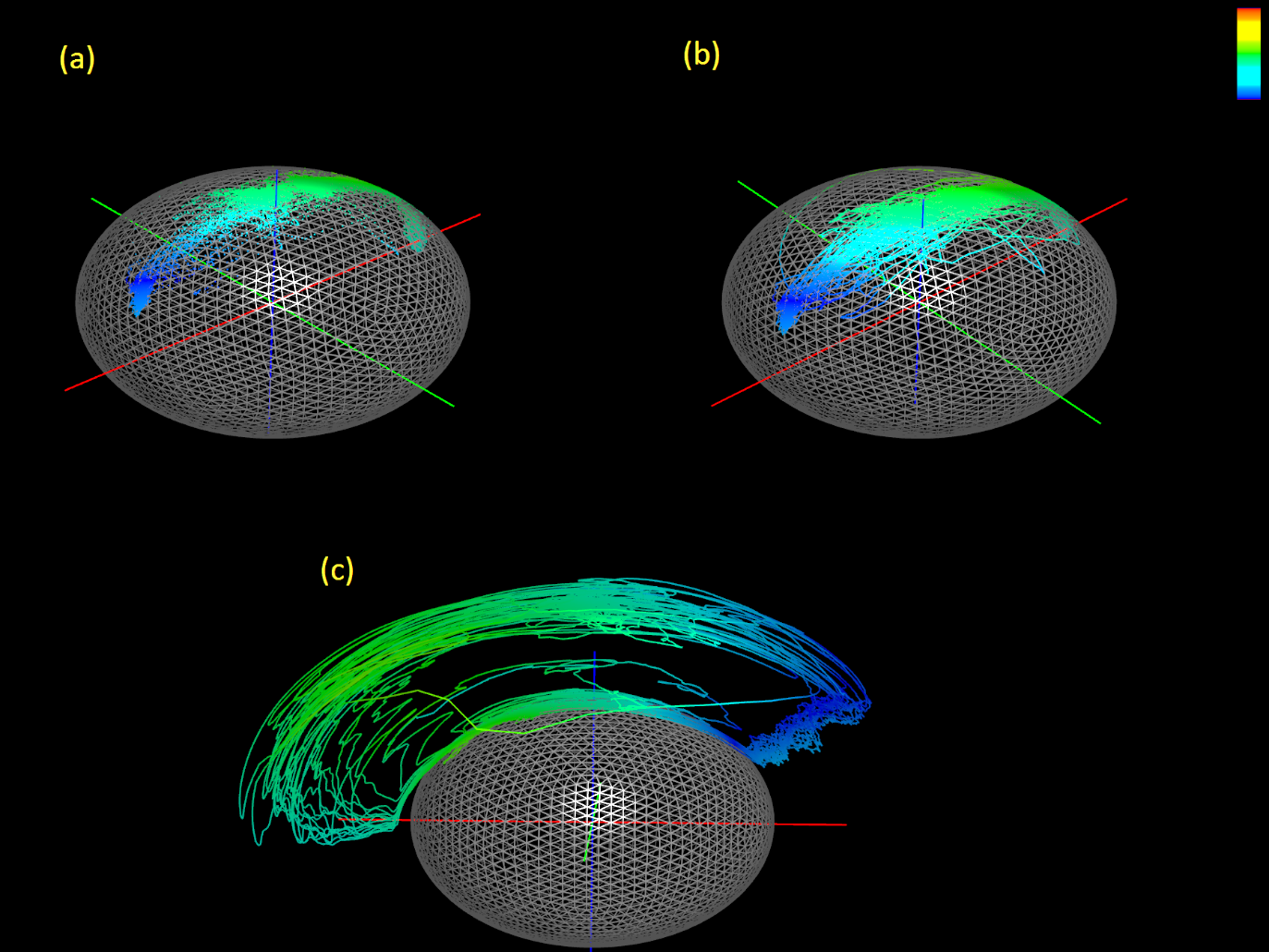


(A)

(B)

(C)

***Fig. S2*** *– A spherical coordinate’s visualisation of* ***(A)*** *postural state plotted onto the surface of a sphere in three-dimensional space.* ***(B)*** *Each point is joined together in chronological order to show the temporal structure of the data.* ***(C)*** *The data are further projected outwards from the sphere according to depth. This shows a compelling visualisation of orientation combined with depth to aid in behaviour analysis.*

***
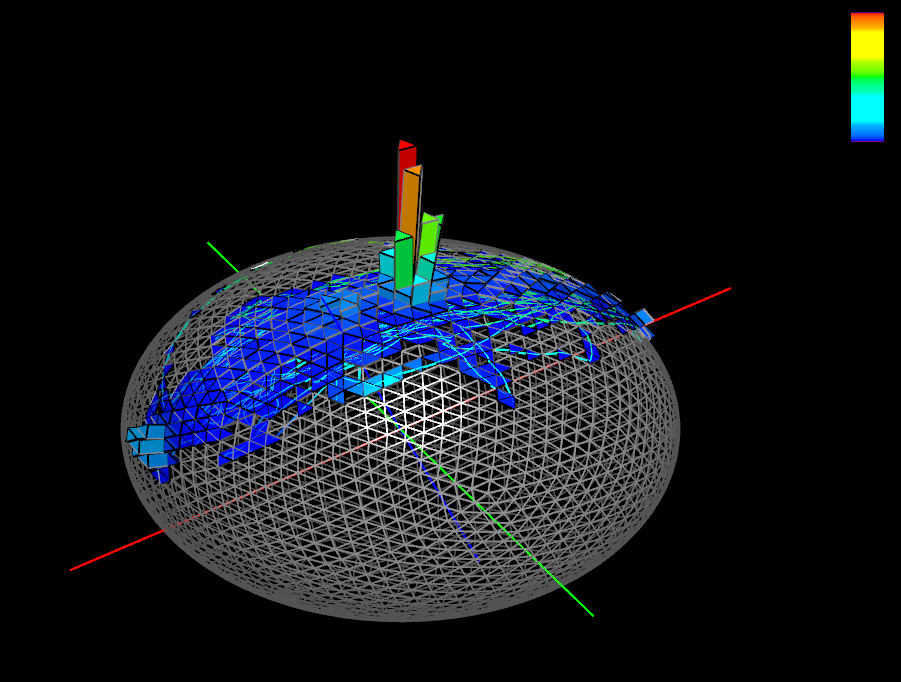
***

***Fig. S3*** *– A spherical histogram (Dubai plot) visualisation to depict frequent postural states. The cormorant spends most time horizontally; on the surface of the water, swimming along the seabed and flying. Variations of the orientation depict less frequent diving and flying patterns.*


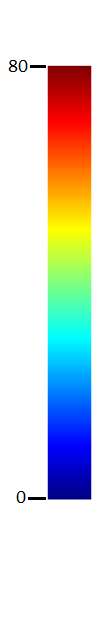

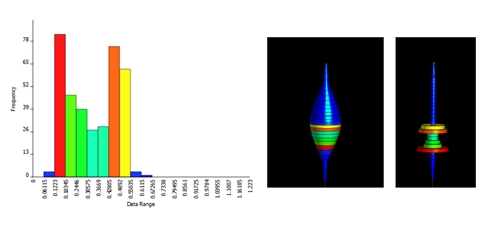


***Fig. S4*** *– Left to right: Histogram, Frequency shape (stacked), fixed shape (skittle). Note the bimodality apparent in the frequency distributions (second left and far right images), which may correspond to different behaviours.*

**Changing shapes for frequency distributions**

(a) Fixed shape (Skittle)

Firstly, a fixed shape spine derived from spinning tops using a Hermite spline^9^ creates a smooth curve. The start and end points of the spline are fixed at the respective top and bottom centre of the spine, with control points leading orthogonally outwards at a radius defined by the facet frequency. The resulting spline is revolved through 360 degrees around the spine axis to create a skittle like object. The skittle is split into segments defined by the number of bins, with each segment colour coded accordingly to the histogram size as per the corresponding bin (Fig. 5 (top)).


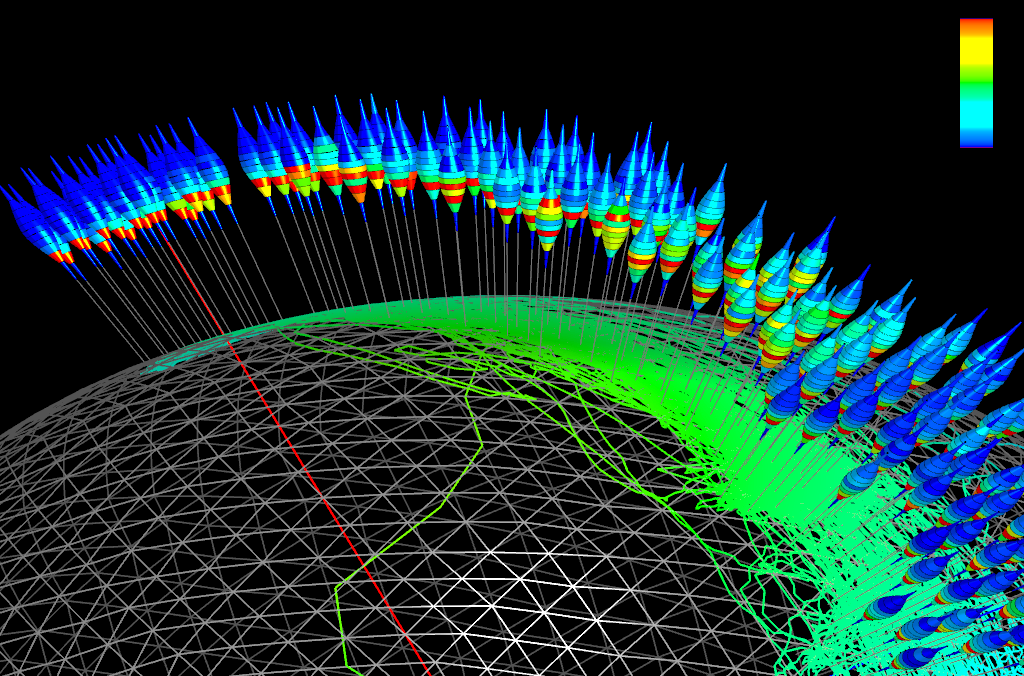

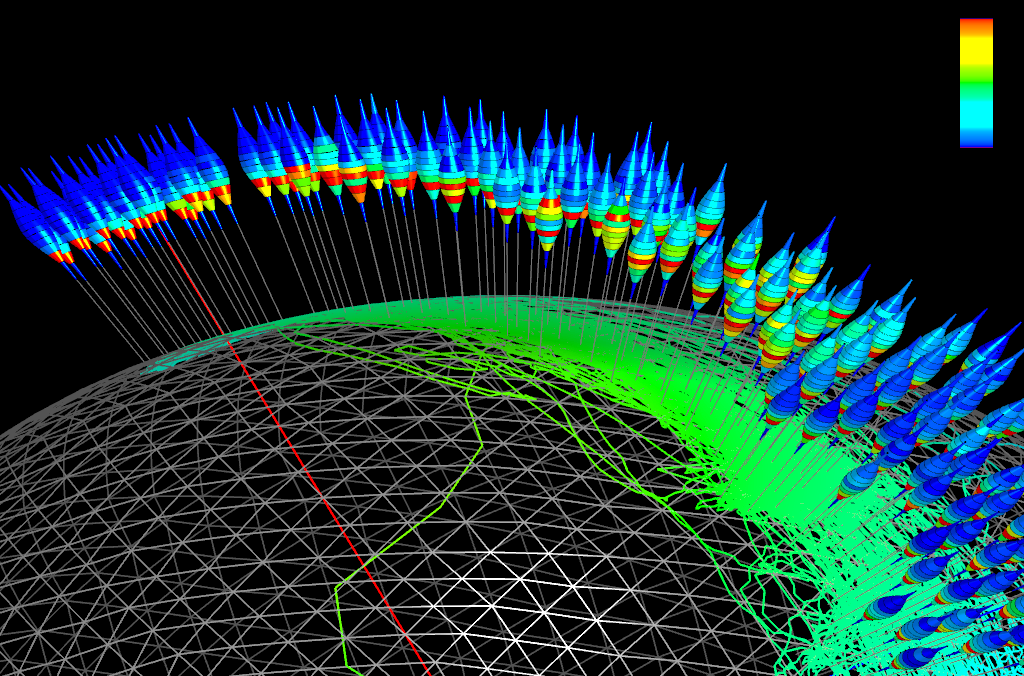


(A)

(B)

***Fig. S5*** *– G-Urchin of skittle shape* ***(A)****, and stacked frequency* ***(B)*** *urchins emitted from the centre of each facet of the sphere. Each urchin represents the underlying data within the corresponding facet. The size of each histogram bin is colour-coded accordingly.*

(b) Frequency shape (Stacked)

Another option is to treat each bin in the histogram as a cylinder, with a fixed height corresponding to bin width, but for which the diameter corresponds to the number, or percentage, of elements inside the corresponding bin. Each cylinder is stacked and the resulting height and width scaled by item frequency which is then colour-coded appropriately. The resulting visualisation allows the incorporation of power usage and behavioural state together in one visualisation. For example, a highly energetic state will be presented as a large cylinder or red highlight near the top of the urchin (Fig. S5 (bottom)).

**User interface**

The g-sphere user interface is built into Framework4 and made accessible via the tools menu. Framework4 uses a multi-tab interface to allow multiple data sets to be loaded and labelled in the software. The g-sphere operates on the data sets and associated classified behaviours. The g-sphere also uses a multi-tab interface for the same purpose and creates multiple g-spheres from different data, attributes, and operations. Upon creation of a g-sphere, a new tab is created which is named by the user for later reference throughout the software. Each tab represents a separate interface specific for interacting with the corresponding g-sphere. The interface (Fig. S6) is split into three components. At the centre is the g-sphere visualisation panel, with which the user can interact by rotating, moving, and scaling to zoom in and out. In the top right, the histogram panel shows an individual instance of a histogram in two-dimensions when a histogram or urchin is double clicked. In the bottom right of the display, the details of the g-sphere are shown. For a data g-sphere, the behaviour classification grouping for the data are shown. Here, the user can select which behaviours to visualise in the g-sphere, which updates the visualisation instantly.


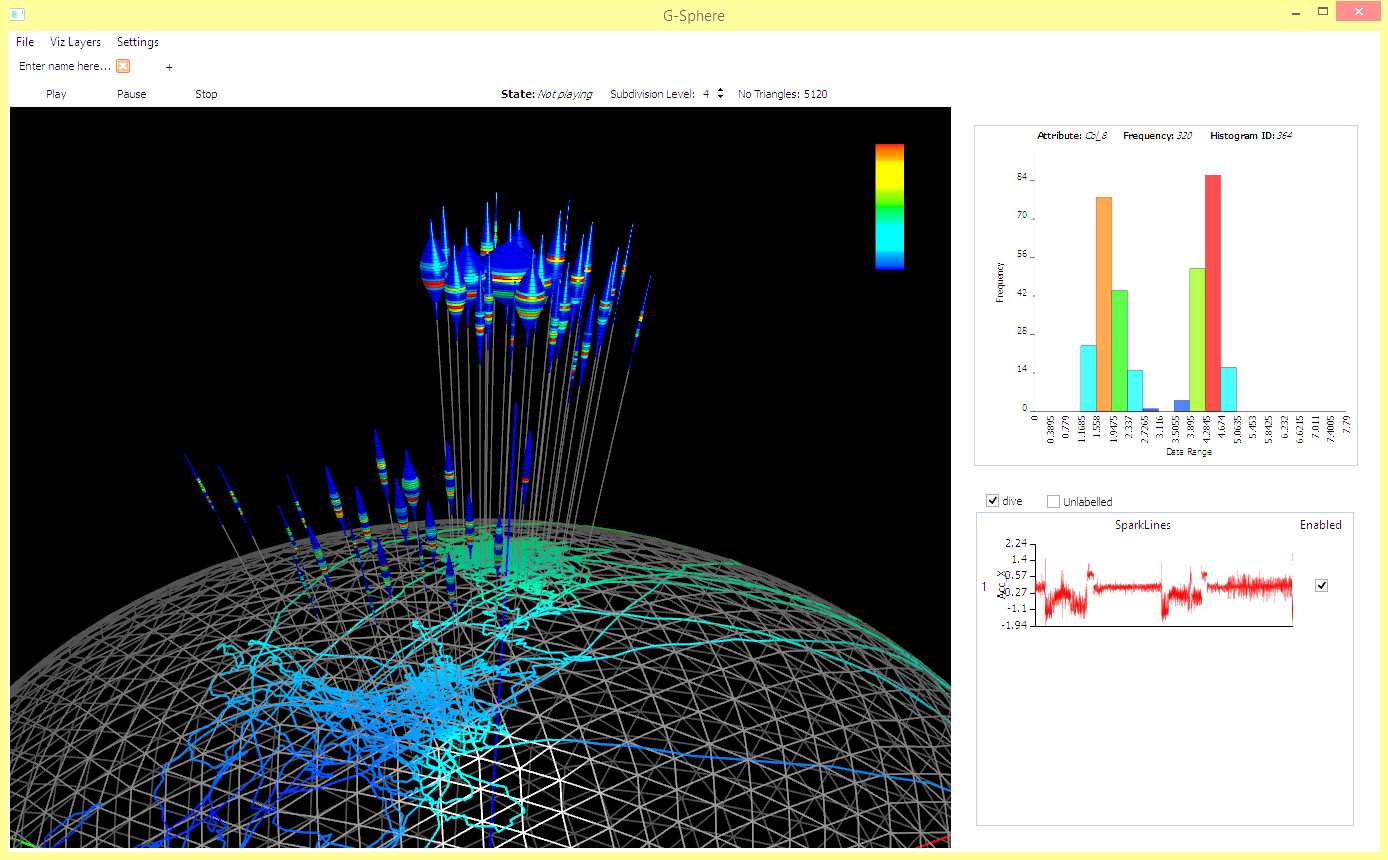


***Fig. S6*** *– Overview of the user interface. Top – Toolbar for constructing a new G-Sphere. Left – Visualisation panel of the G-Sphere. Top Right – Histogram view of a selected g-urchin in the visualisation panel. Bottom Right – Interface to select specific behaviours and data subsets to utilise in the visualisation.*

Menu options allow the user to enable and disable each visualisation technique, along with a settings panel to interact with the visualisations, change bin size and attributes throughout as specified previously. A playback function allows the user to playback the visualisation over time to see how the visualisation evolves. Finally, the user can save the g-sphere workspace and load it back in the work panel at any later date.

***Fig. S7*** *– G-spheres showing periods when an angler was flicking a rod back and forth in preparation for the final cast during fly-fishing with a tri-axial accelerometer attached to the base of the rod. In this case, the radial distance of the points from the sphere was set to be manifest by the vectorial sum of the smoothed acceleration values. During a full casting sequence, the rod is typically flicked backwards and forwards a number of times before the final cast. The cases above show one session consisting of 6 flicks before the final cast and three different sessions, each of 4 flicks before the final cast. Note the differences between sequences, both in the number of flicks and in the variation around the 4-flick casts. Points are separated by 0.025 s.*
